# Sesquiterpene lactones isolated from indigenous Middle Eastern plants inhibit tumor promoter-induced transformation of JB6 cells

**DOI:** 10.1186/1472-6882-12-89

**Published:** 2012-07-09

**Authors:** Melody Saikali, Akram Ghantous, Racha Halawi, Salma N Talhouk, Najat A Saliba, Nadine Darwiche

**Affiliations:** 1 Department of Biochemistry and Molecular Genetics, American University of Beirut, Beirut, Lebanon; 2 Department of Biology, American University of Beirut, Beirut, Lebanon; 3 Ibsar, Nature Conservation Center for Sustainable Futures, American University of Beirut, Beirut, Lebanon; 4 Department of Internal Medicine, American University of Beirut, Beirut, Lebanon; 5 Department of Landscape Design and Ecosystem Management, American University of Beirut, Beirut, Lebanon; 6 Department of Chemistry, American University of Beirut, Beirut, Lebanon

## Abstract

**Background:**

Sesquiterpene lactones (SL) are plant secondary metabolites that are known for their anti-fungal, anti-bacterial, anti-inflammatory, and anti-tumor properties. Considering that several SL-derived drugs are currently in cancer clinical trials, we have tested two SL molecules, 3-β-methoxy-iso-seco-tanapartholide (β-tan) isolated from *Achillea falcata* and salograviolide A (Sal A) isolated from *Centaurea ainetensis*, for their anti-tumor properties. We used the mouse epidermal JB6P + cells as a model for tumor promotion and cellular transformation. Key players that are involved in cellular transformation and tumorigenesis are the AP-1 and NF-κB transcription factors; therefore, we assessed how β-tan and Sal A modulate their signaling pathways in JB6P + cells.

**Methods:**

The effects of β-tan and Sal A on the growth of normal and neoplastic keratinocytes and on the tumor promotion-responsive JB6P + cells were determined using the MTT assay. Anchorage-independent cell growth transformation assays were used to evaluate the anti-tumor promoting properties of these SL molecules in JB6P + cells and dual luciferase reporter assays and western blot analysis were used to investigate their effects on tumor promoter-induced AP-1 and NF-κB activities and protein levels of key AP-1 and NF-кB target genes.

**Results:**

β-tan and Sal A selectively inhibited tumor promoter-induced cell growth and transformation of JB6P + cells at concentrations that do not affect JB6P + and primary keratinocytes basal cell growth. In addition, both molecules reduced basal and tumor promoter-induced NF-κB transcriptional activities, differentially regulated basal and tumor promoter-induced AP-1 transcriptional activities, and modulated key players of the AP-1 and NF-κB signaling pathways.

**Conclusions:**

These results highlight the anti-tumor promoting properties of β-tan and Sal A. These SL molecules isolated from two plant species native to the Middle East may provide opportunities for complementary medicine practices.

## Background

There is a renewed interest in the use of natural compounds to prevent/treat several types of diseases including cancer and inflammatory conditions [1,2]. Currently, there are more than 200 natural product-derived drugs already in preclinical/clinical development or in the clinic [1,3,4]. The therapeutic properties of medicinal plants are generally attributed to plant secondary metabolites, an example of which are sesquiterpene lactones (SL), which are present almost exclusively in plant species belonging to the family *Asteraceae*[5,6]. This family comprises plant species commonly used in ethnomedicine [7], some of which have been reported to specifically treat diseases such as cancer, inflammation, headaches, and infections [6,8]. Sesquiterpene lactones often colorless and with a bitter taste, are a stable form of terpenoids and are divided into four groups: germacranolides, eudesmanolides, guaianolides, and pseudoguaianolides [6]. The bioactivity of a SL molecule has been attributed to several factors including the number of alkylating centers, the lipophilicity of the molecule, and its geometry [9]. Importantly, several SL-derived drugs are currently being tested in cancer clinical trials [9].

Following bioassay guided fractionation, we have isolated, identified, and characterized two SL molecules of the guaianolide group, 3-β-methoxy-iso-seco-tanapartholide (β-tan) and salograviolide A (Sal A), with promising anti-tumor and anti-inflammatory activities [10-14].

β-tan which was purified from *Achillea falcata*, a species native to Lebanon and the Middle East [15], differentially inhibited the growth of the epidermal human HaCaT cells at non-cytotoxic concentrations to primary epidermal keratinocytes [11]. Sal A, which was isolated from *Centaurea ainetensis*, also a species native to Lebanon and the Middle East, was found to possess anti-inflammatory [13,14,16] and anti-cancer activities in a mouse colon cancer model and in skin cancer cells at different stages of tumorigenesis [10,12,17].

In this study, we specifically investigated whether these SL molecules target the tumor promotion stage of tumorigenesis and cell transformation using the well-established JB6 mouse epidermal cell system, which includes the promotion-sensitive P + cells [18,19]. In contrast to tumor initiation, tumor promotion is largely reversible, dependent on epigenetic mechanisms, and is a rate-limiting step in multi-stage carcinogenesis, making it an attractive target for anticancer drugs [20,21]. The JB6P + cells can be transformed to malignancy by tumor promoters, and hence, constitute an ideal model to identify anti-tumor promoting and chemopreventive agents and to decipher their mechanism of action [19,22-24].

The anti-tumor promoting activities of β-tan and Sal A and their modulation of AP-1 and NF-κB signaling were investigated using JB6P + cells. AP-1 and NF-κB signaling pathways have been shown to be up regulated and to play key roles in tumor promotion and epidermal tumorigenesis [19,25]. Members of the AP-1 and NF-κB complexes are expressed at high levels in JB6P + cells [19], and AP-1 and NF-κB activities are required for tumor promotion [26,27]. The inhibition of NF-κB and/or AP-1 activities abrogates transformation in JB6 cells in transgenic mice and in human keratinocytes [25,28-30].

## Methods

### Cells and culture conditions

Primary mouse keratinocytes (PMKs) were freshly prepared from one- to-two day-old neonatal BALB-c mice as described by Yuspa *et al.*[31]. The SP-1 benign tumor cell lines were produced in SENCAR mice [31]. The neoplastic PAM212 cell line is a differentiated squamous cell carcinoma (SCC) that spontaneously transformed *in vitro*[32]. I7 is a spindle cell line derived from a skin carcinoma formed from PMKs infected with the v-*rasHa* and c-*fos* oncogenes and grafted to nude mice [32]. PAM212, SP1, and I7 cell lines were generously provided by Dr. Stuart H. Yuspa (NIH, Bethesda, MD). The JB6P + cell line is a tumor promoter-sensitive clonal variant (clone 41, subclone 5a), derived from the JB6 model for tumor promotion, and originally derived from primary mouse epidermal cells [33]. The JB6P + cell line was generously provided by Dr. Nancy Colburn (NCI, Frederick, MD).

SP1, PAM212, and PMK cells were cultured in fresh Eagle Minimum Essential Medium (EMEM) (Bio Whittaker, Cambrex Co., MD) containing 10% chelated fetal bovine serum (FBS) with no more than 0.05 mM Ca^++^ to maintain a basal proliferating cell phenotype [34], 1% L-glutamine, and 1% penicillin-streptomysin antibiotics (Gibco-BRL Life Technologies, Carlsbad, CA). I7 cells were cultured in complete EMEM medium with 10% FBS, 2 mM L-glutamine, and 1% penicillin-streptomysin. JB6P + cells were cultured in EMEM (SIGMA, M2279) containing 4% heat inactivated FBS (Gibco BRL Life), 2 mM L-glutamine, 25 *μ*g/mL of gentamicin (SIGMA SG 1397 M10) and 1% non-essential amino acids (NEAA) (Gibco). JB6P + cells were used up to ten passages in culture to avoid spontaneous transformation *in vitro*. All cells were grown in a humidified incubator which was set at 95% air and 5% CO_2_ except for PMKs which were grown in 93% air and 7% CO_2_.

### Sesquiterpene lactones isolation and cell treatments

Extraction, purification, and identification of the SL β-tan and Sal A from *Achillea falcata* and *Centaurea ainetensis*, respectively, were performed as previously described [11,14]. Briefly, the plant material was soaked in methanol and then subjected to filtration and several fractionation steps where the different fractions were subjected to bio-guided fractionation. The sub-fractions with the most potent anti-proliferative activities were further purified, and the pure bioactive compounds, Sal A from *Centaurea ainetensis* and β-tan from *Achillea falcata* were identified using ^1^ H and ^13^ C NMR identified using several spectroscopic techniques including 1D and 2D NMR as well as mass spectrometry, UV, and IR. β-tan and Sal A were prepared from a stock of 20 mg/ml diluted in absolute ethanol. Cells were treated with the indicated concentrations of β-tan and Sal A. For the control conditions, concentrations of ethanol in culture medium did not exceed 0.1% which had no effect on the growth of cells (data not shown).

### Cell growth assay

Cell growth was assayed at indicated time points using the MTT Cell Proliferation Kit according to manufacturer’s instructions (Roche Diagnostics). The proliferation assay is an MTT-based method which measures the ability of metabolically active cells to convert tetrazolium salt into a blue formazan product, the absorbance of which is recorded at 595 nm using an ELISA microplate reader. Cell growth results were expressed as percentage of control and were derived from the mean of triplicate wells.

Cells were seeded in 96-well plates, at a density of 1 x 10^5^ cells/ml in 100 μl media, and incubated until confluency reached 50%. After which the media was removed and 100 μl of fresh media containing different concentrations of β-tan or Sal A were placed for treatment conditions, or a maximum of 0.1% ethanol in media for control conditions. For MTT assays using the phorbol ester 12-O-tetradecanoylphorbol-13-acetate (TPA) (Enzo Life Sciences, USA), JB6P + cells were treated with either 5 nM TPA [35] in media only, or with the indicated concentrations of β-tan or Sal A with or without 5 nM TPA co-treatment.

### Anchorage-independent growth transformation assay

Colony growth in soft agar is a well-established index of cell transformation [24]. Anchorage-independent growth was studied using the CytoSelect^TM^ 96-Well Cell Transformation Assay kit (Cell Biolabs) according to manufacturer’s instructions. The base agar layer (0.6% agar, 10% FBS, 2 mM L-glutamine and 25 μg/ml gentamicin) was layered into wells of a 96-well plate and allowed to solidify. Once solidified, the cell agar layer containing 0.4% agar with JB6P + cells treated with the indicated concentrations of β-tan and Sal A, with 5 nM TPA [23,35,36], in complete EMEM (10% FBS), was layered on top of the base agar layer. The indicated concentrations of β-tan and Sal A were then prepared in complete EMEM (10% FBS), with 5 nM TPA and placed over the solidified cell agar layer. The cells were incubated for 9 ± 1 day at 37°C and 5% CO_2_, replenished with the indicated concentrations of β-tan and Sal A with 5 nM TPA every 3 days. Colonies were photographed and then quantified using the CyQuant GR Dye where the fluorescence was measured using a 96-well fluorometer set at a 485/520 nm filter set.

### Dual luciferase reporter assay for AP-1 and NF-κB transcriptional activities

JB6P + cells were seeded in 24-well plates (1 x 10^5^ cells/ml), and at 60–80% confluency, cells were co-transfected with the AP-1 (pXP2-35alb-Luc, 0.8 μg) or NF-κB (pGL2-IL-6–Luc, 0.8 μg) *firefly* luciferase reporter plasmids with the *renilla* luciferase reporter plasmid (pRL-SV40, 0.04 μg). The pXP2-35alb-Luc harbors the albumin promoter upstream from the luciferase gene. Within this promoter, the GCN4 oligo sequence, which harbors the AP-1 binding site, was ligated. The pGL2-IL-6–Luc uses the IL-6 promoter region containing four putative NF-κB binding sites. These reporter plasmids were kindly provided by Dr. Nancy Colburn (NCI). Co-transfection was done using Lipofectamine^TM^ 2000 with PLUS^TM^ reagent (Invitrogen), without antibiotics for 3 h at 37°C, 5% CO_2_, then replenished with complete EMEM (4% heat-inactivated FBS, with antibiotics) for at least 12 h. Cells were then treated with the indicated concentrations of β-tan and Sal A, with or without 16 nM TPA for 24 h as described [35]. Cell lysates were then prepared and luminescence measured using the Dual Luciferase Reporter Assay Kit (Promega) as per manufacturer’s instructions. The *firefly* reporter transfection efficiencies were normalized relative to the *renilla* luciferase activity generated by this vector and plotted as percentage of control.

### Western blot analysis

JB6P + cells were plated in 100 mm dishes at a density of 50,000 cells/ml. At 80–90% confluency, cells were starved with 0.1% FBS for 24 h, then were pre-treated with either 10 μg/ml β-tan or 15 μg/ml Sal A for 1 hr followed by 15 min or 6 h 32 nM TPA [35,37]. Whole cell protein extracts (30 μg) were prepared as described [10] and probed overnight at +4 °C with primary antibodies against MMP-9 (Chemicon, Millipore) MMP-2 (Chemicon, Millipore), GAPDH, IκBα, cyclin D_1_, p16, Bax and Bcl-2 (Santa Cruz Biotechnology, Inc.) followed by secondary antibodies conjugated with horseradish peroxidase. Equal protein loading and quality were verified through GAPDH reprobing and Ponceau staining of membranes. The immunocomplexes were visualized using enhanced chemiluminescent kits obtained from Santa Cruz (ECL system). Bands were quantified using ImageQuant software and the Molecular Dynamics 860 System (Molecular Dynamics, Sunnyvale, CA). In some western blots, adjustments of brightness and contrast were applied to all bands of the same membrane image.

### Statistical analysis

Data presented are the means ± SE of at least two independent experiments or as indicated. Significant differences were determined using the *post-hoc* tests; Tukey, SNK and Dunnett tests of the SPSS Version 16.0 software. Significance was set at indicated p-values (0.05, 0.01 or 0.001).

## Results

**Figure 1:**
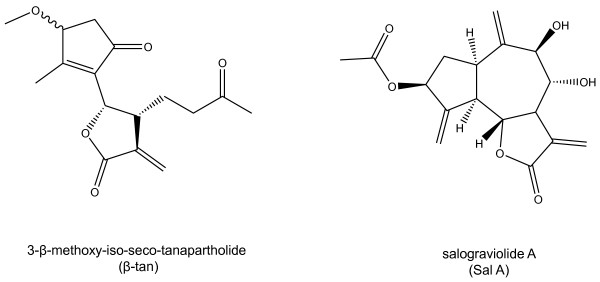
** Chemical structures of 3-β-methoxy-iso-seco-tanapartholide (β-tan) and salograviolide A (Sal A) from*Achillea falcata*and*Centaurea ainetensis*, respectively.**

We have previously shown that β-tan and Sal A which belong to the same guaianolide group, exhibit selective anti-tumor activities with minimal effects on normal cells [11,17]. In this study, we investigated whether Sal A and β-tan (Figure 1), attenuate tumor promotion, using the JB6 tumor model. We focused on AP-1 and NF-κB signaling pathways, known to play crucial roles in tumor promotion and in epidermal carcinogenesis [19].

### β-tan and Sal A selectively inhibit the growth of tumor cells

**Figure 2:**
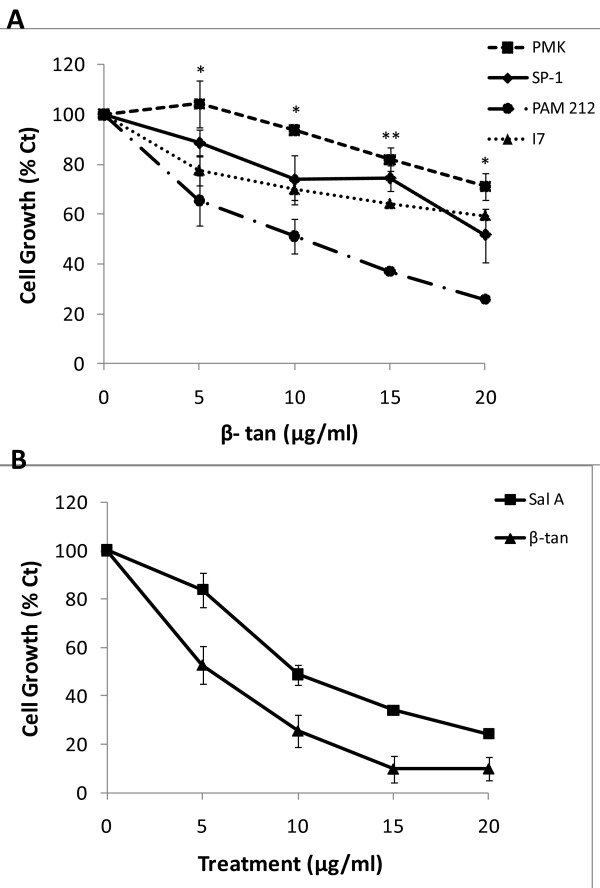
** β-tan and Sal A selectively inhibit the growth of JB6P+ cells.** At 50–60% confluency, primary mouse keratinocytes (PMK), SP-1, PAM212, I7 (A) and JB6 P+ (B) cells were treated with the indicated β-tan or Sal A concentrations, or 0.1% ethanol as the control. Cell growth was determined at 24 h and expressed as percentage of control (Ct) treated cells using the MTT Cell Proliferation Kit, as described in Materials and Methods. Significance between neoplastic cell lines and PMKs is indicated by * at *p* < 0.05 or ** at *p* < 0.01. Results represent the mean (± SEM) of at least two independent experiments done in triplicate wells.

We have previously shown, in a murine *in vitro* model of epidermal carcinogenesis, that Sal A selectively inhibits the cell growth of papilloma and SCC cell lines without significantly affecting the growth of normal cells [10]. Here, we characterized the growth-inhibitory effects of β-tan *in vitro* using an MTT-based assay. In this model, the primary mouse keratinocytes (PMKs) are representatives of normal cells, the SP-1 cell line as benign tumor cells, PAM-212 cell line as SCC, and the spindle I7 cells as aggressive and metastasizing tumor cells. Treatment with β-tan caused a dose-dependent growth inhibition at 24 h, where a concentration of 10 μg/ml decreased cell growth significantly by 49 ± 7% (*p* < 0.01) in PAM 212 cells compared to a 6 ± 1% decrease in PMKs cell growth (Figure 2A). The benign SP-1 cells and spindle I7 cells appeared to be less sensitive at this concentration, showing a 26 ± 10% and 30 ± 4% decrease, respectively, that were not significantly different than the normal PMKs (Figure 2A). We have previously performed similar experiments on Sal A and found that 10 μg/ml is selective for tumor cells [10]. In this study, we used this same concentration (10 μg/ml) to study the effect of both β-tan and Sal A on JB6P + cell growth and transformation. β-tan and Sal A produced a dose-dependent growth inhibition in JB6P + cells (Figure 2B). Treatment with 10 μg/ml β-tan and Sal A inhibited JB6P + cell growth by a significant 74 ± 7% and 51 ± 4% (*p* < 0.01), respectively (Figure 2B). These results show that at low concentrations, both molecules preferentially inhibited the growth of JB6P + cells *versus* normal keratinocytes, eliminating the possibility that the anti-tumor promoting effects of β-tan and Sal A is due to drug cytotoxicity.

### β-tan and Sal A inhibit tumor promoter-induced proliferation and transformation of JB6P + cells

**Figure 3:**
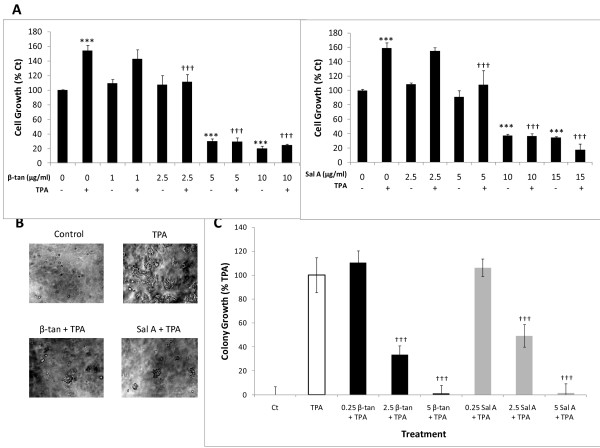
** β-tan and Sal A inhibit promoter-induced proliferation and transformation of JB6P + cells.** At 50–60% confluency, JB6P + cells were treated with the indicated concentrations of β-tan and Sal A, with or without TPA co-treatment. **(A)** Cell growth was determined at 48 h and expressed as percentage of control (Ct) treated cells using the MTT Cell Proliferation Kit. Significance from TPA treatment is indicated by ††† at *p* < 0.001 while significance from control treatment is indicated by *** at *p* < 0.001. Results represent the averages (± SEM) of at least two independent experiments done in triplicate wells. **(B, C)** In the anchorage-independent growth assay, JB6P + cells were suspended in 0.4% soft agar over 0.6% base agar layers, with the indicated β-tan and Sal A concentrations (μg/ml) and TPA co-treatment. Treatments were replenished every three days, where the co-treatment of 2.5 μg/ml β-tan or Sal A with TPA and colony growth was photographed at 200X magnification (B) and quantified at 9 ± 1 day post-seeding using the CytoSelect^TM^ Transformation Assay (C). Colony growth is expressed as percentage of TPA only-treated cells and plotted as mean colony growth (± SEM) of two independent experiments done in triplicate wells (C). Significance from TPA control is indicated by ††† at *p* < 0.001.

We investigated the anti-tumor promoting properties of β-tan and Sal A in JB6P + cells. Tumor promoters, such as the phorbol ester 12-*O-*tetradecanoylphorbol-13-acetate (TPA), increase JB6P + cell growth and transformation. Treatment of JB6P + cells with TPA alone significantly increased their growth at 48 h by approximately 160 ± 7% relative to control (*p* < 0.001) (Figure 3A). However, co-treatment with β-tan or Sal A with TPA for 48 h inhibited tumor promoter-induced proliferation of JB6P + cells (Figure 3A). β-tan treatment for 48 h at 1 or 2.5 μg/ml did not cause a significant growth inhibition of JB6P + cell proliferation compared to control treated cells (*p* > 0.05) (Figure 3A). However, co-treatment of 2.5 μg/ml β-tan with TPA showed a significant (*p* < 0.001) inhibition of TPA-induced proliferation, by 28 ± 10%, relative to the TPA-treated cells; whereas, co-treatment of 1 μg/ml β-tan with TPA showed no significant inhibition on TPA-induced proliferation (*p* > 0.05) (Figure 3A). β-tan concentrations of 5 and 10 μg/ml had a significant growth inhibitory effect after 48 h on JB6P + cells (by 70 ± 3% and 80 ± 3% respectively) relative to control (*p* < 0.001), and when co-treated with TPA, cell proliferation was significantly decreased (*p* < 0.001) (Figure 3A).

Treatment with Sal A at 5 μg/ml had no growth inhibitory effect in JB6P + cells while this concentration caused a significant inhibition of TPA-induced proliferation by 33 ± 20% relative to the TPA-treated cells (*p* < 0.001) (Figure 3A). Higher concentrations of Sal A at 10 or 15 μg/ml caused a significant 63 ± 3% and 65 ± 1% decrease in cell proliferation, respectively, with or without the presence of TPA (*p* < 0.001) (Figure 3A). These results indicate that both SL molecules reduced tumor promoter-induced proliferation of JB6P + cells at concentrations that did not affect the growth of normal cells.

To test whether these two SL molecules inhibit tumor promoter-induced cell transformation, we determined their effects on anchorage-independent cell growth in soft agar, which is a hallmark of malignant transformation. In the presence of tumor promoters, the immortalized but non-tumorigenic JB6P + cells become tumorigenic, forming colonies in an anchorage-independent manner [23]. JB6P + cells treated with only TPA, but not solvent control, exhibit colony growth in soft agar (Figure 3B, C). Importantly, upon co-treatment of β-tan or Sal A with TPA, colony formation was inhibited in a concentration-dependent manner in JB6P + cells (Figure 3B, C). At 0.25 μg/ml, neither β-tan nor Sal A decreased JB6P + colony growth 9 ± 1 day after seeding; however, at 2.5 μg/ml concentrations, which were non-cytotoxic to normal and JB6P + cells by MTT (Figure 2), β-tan and Sal A significantly inhibited tumor promoter-induced colony formation by 66 ± 8% and 51 ± 8%, respectively (*p* < 0.001) (Figure 3B, C). Both SL molecules completely abrogated colony growth 9 ± 1 day post-seeding at 5 μg/ml concentrations. These results show that β-tan and Sal A inhibit tumor promoter-induced JB6P + cell transformation.

### β-tan and Sal A differentially modulate TPA-induced NF-κB and AP-1 activities in JB6P + cells

Elevated levels of AP-1 and NF-κB activities are hallmarks of malignant transformation [19,27,37]. Since β-tan and Sal A both inhibited tumor promoter-induced cell transformation, we hypothesized that these SL molecules mediate their anti-tumor promoting activities by repressing AP-1, NF-κB, or both transcriptional activities.

**Figure 4:**
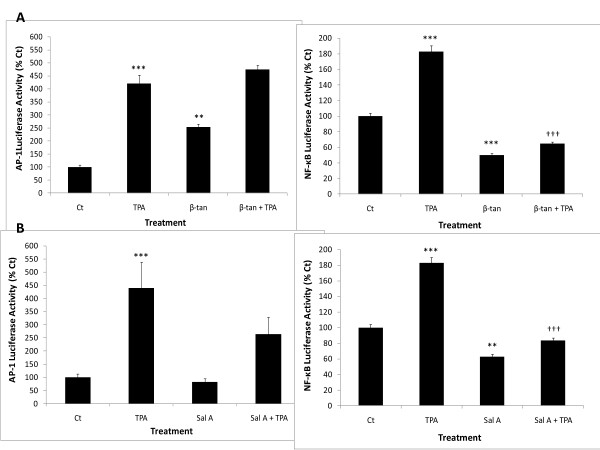
** β-tan and Sal A decrease basal and TPA-induced NF-κB transcriptional activities.** At 60–80% confluency, JB6P + cells were transiently co-transfected with control *renilla* luciferase reporter construct and AP-1 or NF-κB *firefly* luciferase reporter constructs and were pre-treated with the indicated concentrations of β-tan** (A)** or Sal A **(B)** for 1 h followed by TPA treatment up to 24 h. Results were standardized relative to *renilla* luciferase and expressed as percentage of control (Ct)-treated cells. Significance from Ct-treated condition is indicated as ** at *p* < 0.01 and *** at *p* < 0.001. Significance from TPA-control condition is indicated as †† at *p* < 0.01 and ††† at *p* < 0.001. Results are representative of two independent experiments and plotted as averages of triplicate wells (± SD).

The application of TPA alone dramatically increased AP-1 and NF-κB luciferase activities in JB6P + cells by four- and approximately two-fold, respectively, compared to control (Figure 4). We tested the effects of β-tan and Sal A on TPA-induced AP-1 and NF-κB transcriptional activities for 24 hours, using 5 μg/ml concentrations as these completely abrogated colony formation with minimal effects on primary keratinocyte cell growth. Unexpectedly, at this concentration, β-tan showed a significant 2.5-fold increase in basal AP-1 activity, relative to control (*p* < 0.01) and did not decrease TPA-induced AP-1 activity (Figure 4A). Importantly, 5 μg/ml β-tan showed a significant inhibition of basal and TPA-induced NF-κB activity by 50 ± 4% and 64 ± 4%, respectively, at 24 h (*p* < 0.001) (Figure 4A).

Sal A (5 μg/ml) did not modulate basal AP-1 activity, but caused a non-statistically significant decrease in TPA-induced AP-1 activity. Interestingly, Sal A significantly decreased basal and TPA-induced NF-κB transcriptional activities at 24 h by 37 ± 6% and 54 ± 5%, respectively (*p* < 0.01) (Figure 4B). Our experiments show that both β-tan and Sal A decreased basal and tumor promoter-induced NF-κB activities, which in fact is a characteristic property of SL [6].

### β-tan and Sal A modulate key target genes of the AP-1 and NF-κB signaling pathways in JB6P + cells

**Figure 5:**
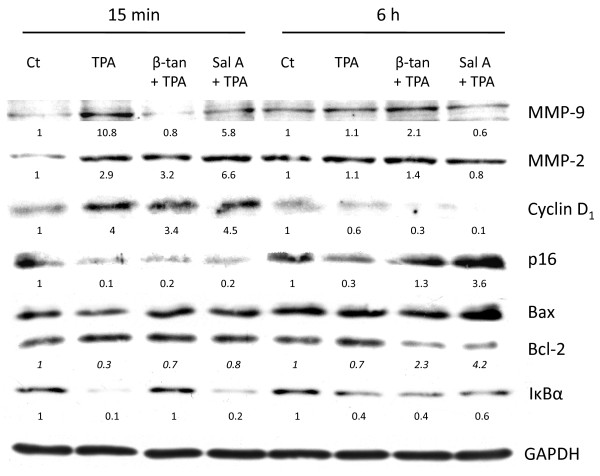
** β-tan and Sal A modulate the protein levels of key AP-1 and NF-κB downstream target genes in JB6P + cells.** JB6P + cells were plated in 100 mm dishes at a density of 50,000 cells/ml. At 80–90% confluency, cells were starved with 0.1% FBS for 24 h, then were pre-treated with either 10 μg/ml β-tan or 15 μg/ml Sal A for 1 h, followed by 15 min or 6 h TPA treatment. Whole cell proteins were immunoblotted with the indicated antibodies and reprobed with GAPDH antibody to ensure equal protein loading. Densitometry values were standardized relative to GAPDH for each condition and results are expressed relative to control (Ct) treated cells for each time point. Densitometry values for Bax:Bcl-2 protein ratios are indicated in *italics*.

In JB6 cells, both AP-1 and NF-κB activities are essential for the transformation response, which can be attributed to their roles in the transcriptional activation of genes controlling cellular proliferation, metastasis, angiogenesis, tumor invasion, and apoptosis [38,39]. We next investigated the effect of β-tan and Sal A on the protein levels of key downstream targets of the AP-1 and NF-κB pathways known to be induced by tumor promoters in cell transformation and tumor progression. These target genes are modulated by tumor promoters at early time points; therefore, we pretreated JB6P + cells for one hour with high concentrations of β-tan (10 μg/ml) and Sal A (15 μg/ml), followed by TPA for 15 minutes or 6 hours. We chose these high concentrations that kill approximately 70% of cells by 24 h to be able to detect early protein changes of key AP-1 and NF-κB target genes. Protein levels of metalloproteinase 9 (MMP-9) were induced by approximately 11-fold in TPA-treated JB6P + cells as early as 15 minutes and were reduced to basal levels and by approximately 50% by pre-treatment with β-tan and Sal A, respectively (Figure 5). On the other hand, MMP-2 protein levels were induced by three-fold in TPA-treated JB6P + cells at 15 minutes but were not reduced by β-tan or Sal A pretreatment. As early as 15 minutes post-TPA treatment, cyclin D_1_ protein levels were increased by four-fold, and were slightly decreased upon pretreatment with β-tan (Figure 5). The cyclin-dependent kinase inhibitor (CDKI) p16 was reduced by TPA at 15 minutes and 6 hours, and pretreatment with β-tan or Sal A increased p16 protein levels to control or higher levels by 6 hours (Figure 5). Furthermore, we investigated the changes in pro-apoptotic Bax and anti-apoptotic Bcl-2 proteins upon treatment with β-tan or Sal A in the presence of TPA. These apoptotic regulators are also key target genes for mediating the AP-1 and NF-κB transformation response. An increase in the ratio of pro-apoptotic over anti-apoptotic Bcl-2 proteins leads to an increase in mitochondrial permeability and subsequent release of cytochrome c, an event central to apoptotic activation [40]. Treatment with TPA alone reduced the pro-apoptotic Bax/Bcl-2 protein ratio to 0.3 folds of control as early as 15 minutes (Figure 5). Pre-treatment with β-tan or Sal A restored the Bax/Bcl-2 protein ratio to almost control values at 15 minutes and to more than two- and four-fold of control values at 6 hours post-TPA treatment (Figure 5).

Since both SL molecules inhibited TPA-induced NF-κB transactivation, we next studied their effects on the NF-κB inhibitor, IκBα. Treatment with TPA alone abrogated IκBα protein levels as early as 15 minutes (Figure 5). Interestingly, only pre-treatment with β-tan restored IκBα protein levels after 15 minutes of TPA-treatment. These results indicate that pretreatment with β-tan or Sal A regulate TPA-induced AP-1 and NF-кB target genes that are involved in the regulation of cell growth, cell migration, and metastasis.

## Discussion

In this study, we investigated the anti-tumor promoting effects of β-tan and Sal A, isolated from *Achillea falcata* and *Centaurea ainetensis,* respectively, using the JB6 epidermal cell model of tumor promotion and cell transformation. In the multi-stage model of carcinogenesis, the tumor promotion phase is a rate limiting step that is responsible for the clonal expansion of initiated cells and is largely reversible [41], offering a practical approach for identifying potential inhibitors of cancer development [42].

Herein, we report that treatment with either Sal A or β-tan preferentially inhibited the growth of murine neoplastic keratinocytes, whilst sparing normal cells. The promotion-sensitive JB6P + cells were the most sensitive to β-tan treatment at concentrations that did not affect the growth of PMKs. Treatment with Sal A was relatively less potent on JB6P + cells, compared to β-tan, where 10 μg/ml β-tan inhibited cell growth by 74 ± 7%, whereas 10 μg/ml Sal A inhibited by 51 ± 4%. Although both belong to the SL guaianolide family, it seems that β-tan, with its relatively open ring structure, possesses higher flexibility, possibly enhancing β-tan diffusion across the cell membrane; in contrast to Sal A which bears a closed ring structure (Figure 1). In addition to the bioactive –α-methylene-γ-lactone ring present in Sal A and β-tan, the latter harbors an additional alkylating center, the cyclopentenone. Moreover, the presence of two hydroxyl (OH) groups within Sal A renders the molecule less lipophilic, possibly decreasing cell membrane penetration and may explain its reduced toxicity to JB6P + cells compared to β-tan.

In studying the anti-tumor promoting properties of these two purified SL molecules, it was essential to assess their effect on TPA-induced JB6P + cell transformation. In this study, we found that both β-tan and Sal A inhibited TPA-induced JB6P + cell transformation, at concentrations not cytotoxic to normal nor to the non-tumorigenic JB6P + cells. A hallmark of cell transformation is the ability of malignant cells to grow in soft agar in an anchorage-independent manner [18,23,36]. Our results show that β-tan and Sal A, at concentrations that did not inhibit JB6P + cell proliferation, were effective in reducing TPA-induced proliferation and inhibiting TPA-induced colony formation. These results suggest that β-tan and Sal A may have promising chemopreventive properties in epidermal carcinogenesis. Future *in vivo* experiments are required to confirm the chemopreventive properties of these purified SL molecules. However, a limiting step for *in vivo* studies will be the availability of large quantities of these molecules.

The activation of the transcription factors AP-1 and NF-κB is essential for tumor promotion and neoplastic transformation, and are highly expressed in the promoter-sensitive JB6P + cells, and the inhibition of both or either one of these signaling pathways is sufficient to inhibit neoplastic transformation [19,23,25]. To study the modulation of tumor promoter-induced AP-1 and NF-κB transcriptional activities by β-tan and Sal A in JB6P + cells, concentrations that inhibited JB6P + cell transformation and did not affect normal cell growth were used. Interestingly, both SL molecules decreased basal and TPA-induced NF-κB activities, but not of TPA-induced AP-1 activity. This suggests that β-tan and Sal A primarily inhibit NF-κB signaling in tumor cells. In fact, it is well established that NF-κB is a vital molecular target for various SL, and some of them, such as parthenolide, artimisinin and thapsigargin are currently in cancer clinical trials [6,9,43]. This can be attributed to the presence of the α-methylene-γ-lactone functional group, which directly alkylates cysteine residues of the p65 subunit, interfering with DNA binding [6,44]. In fact, elevated NF-κB signaling is sufficient to induce epidermal tumor transformation [27]. This prompted us to study the effect of these SL molecules on the protein levels of one of the main NF-κB inhibitors, IκBα. Previous studies have shown that the expression of non-degradable mutants of IκBα and antisense RNA inhibition of NF-κB, result in tumor regression [29,45-47]. Interestingly, only pre-treatment with β-tan restored IκBα protein levels after 15 minutes of TPA-treatment, suggesting that Sal A and β-tan differentially mediate their inhibition of NF-κB signaling. This differential regulation of IκBα proteins by the SL molecules can be attributed to their differences in alkylating centers and lipophilicity, thus, affecting their interaction with the IκBα proteins. Nevertheless, β-tan also significantly increased basal AP-1 levels in JB6P + cells at concentrations that decreased cell growth. This may implicate the dual role of AP-1 in increased cell proliferation and cell death [48].

Since earlier studies have shown that AP-1 and NF-κB can interact together [49], we assessed how both SL molecules modulated key downstream target genes, containing TPA response elements (TREs) common to both AP-1 and NF-κB. Metalloproteinases are essential for tumor promotion, progression, and invasion and AP-1 and NF-κB play a dominant role in the transcriptional activation of the majority of MMPs [50,51] including MMP-9 and MMP-2. In fact, it was shown in mice lacking MMP-9 that this gene is functionally involved in the regulation of oncogene-induced keratinocyte hyperproliferation, progression to invasive cancer, and end-stage malignant grade epithelial carcinomas [52]. Treatment of TPA-promoted JB6P + cells with β-tan or Sal A, abrogated MMP-9, but not MMP-2, protein levels. This implies that the two SL molecules differentially modulate MMP protein levels suggesting the regulation of MMP2 by factors other than AP-1 and NF-κB.

Another important AP-1 and NF-κB target gene is the CDKI p16. Both SL molecules noticeably up regulated p16 that was reduced upon TPA treatment, which suggests that β-tan and Sal A inhibit cell cycle progression that is induced by tumor promoters. Furthermore, AP-1 and NF-κB components also regulate apoptotic proteins such as the pro-apoptotic Bax and the anti-apoptotic Bcl-2 proteins [38,51]. SL are known to be inducers of apoptosis in a variety of cancer cells, and this is considered one of the important mechanisms by which SL exert their anti-tumor properties [6]. Our results show that both β-tan and Sal A increase the Bax:Bcl-2 ratios in TPA-promoted JB6P + cells and suggest that Bcl-2 family members are involved in the growth suppressive effects of β-tan and Sal A.

## Conclusions

This is the first report which investigates the anti-tumor promoting effects of the SL β-tan and Sal A in cell transformation. Our studies highlight the mechanism by which these SL molecules inhibit tumor promotion by reducing TPA-induced NF-κB activity and in regulating several downstream players involved in cell cycle progression, apoptosis, and tumor invasion. It is well established that tumor promotion is epigenetically regulated, and numerous plant-derived anti-cancer drugs are modulators of epigenetic processes [53], therefore it would be interesting to test whether these purified SL molecules are epigenetic regulators. Finally, future studies investigating the anti-tumor promoting properties *in vivo* are needed to test the potential chemopreventive use of these SL molecules.

## Abbreviations

AP-1, Activator protein-1; β-tan, 3-β-methoxy-iso-seco-tanapartholide; CDKI, Cyclin dependent kinase inhibitor; EGF, Epidermal growth factor; EMEM, Eagle’s minimum essential medium; FBS, Fetal bovine serum; IκBα, Inhibitor of NF-κB; MMP, Matrix metalloproteinase; NF-κB, Nuclear factor-κB; NEAA, Non-essential amino acids; NMR, Nuclear magnetic resonance; PMKs, Primary mouse keratinocytes; Sal A, Salograviolide A; SEM, Standard error of mean; SL, Sesquiterpene lactones; TNFα, Tumor necrosis factor α; TPA, 12-O-tetradecanoylphorbol-13-acetate; TREs, TPA response elements.

## Competing interests

The authors declare that they have no competing interests.

## Authors’ contributions

MS, AG and RH performed the experiments and data analysis. MS wrote the manuscript. AG provided statistical analysis and revised the manuscript. NS supervised the extraction and identification of β-tan and Sal A. SNT contributed in the identification and selection of plant species with potential medicinal properties, provided plant material for isolation and testing of molecules, and contributed to the editing of the manuscript. ND designed and oversaw the study, interpreted the data, and revised the manuscript. All authors read and approved the final manuscript.

## Pre-publication history

The pre-publication history for this paper can be accessed here:

http://www.biomedcentral.com/1472-6882/12/89/prepub

## References

[B1] Harvey AL Natural products in drug discovery Drug Discovery Today 2008 13 894 901 10.1016/j.drudis.2008.07.004 18691670

[B2] Shah B Seth A Maheshwari K A review on medicinal plants as a source of anti-inflammatory agents Journal of Medicinal Plants Research 2011 5 101 115

[B3] Darwiche N El-Banna S Gali-Muhtasib H Cell cycle modulatory and apoptotic effects of plant-derived anticancer drugs in clinical use or development Expert Opinion on Drug Discovery 2007 2 361 379 10.1517/17460441.2.3.361 23484647

[B4] Traditional Medicine Fact Sheet No. 134 2008 http://www.who.int/mediacentre/factsheets/fs134/en/

[B5] Merfort I Perspectives on sesquiterpene lactones in inflammation and cancer Current Drug Targets 2011 12 1560 73 10.2174/138945011798109437 21561425

[B6] Zhang S Won YK Ong CN Shen HM Anti-cancer potential of sesquiterpene lactones: bioactivity and molecular mechanisms Current Medicinal Chemistry-Anti-Cancer Agents 2005 5 239 249 10.2174/1568011053765976 15992352

[B7] Kaij-a-Kamb M Amoros M Girre L Search for new antiviral agents of plant origin Pharmaceutica acta Helvetiae 1992 67 130 147 1438451

[B8] Gurib-Fakim A Medicinal plants: traditions of yesterday and drugs of tomorrow Molecular aspects of Medicine 2006 27 1 93 10.1016/j.mam.2005.07.008 16105678

[B9] Ghantous A Gali-Muhtasib H Vuorela H Saliba NA Darwiche N What made sesquiterpene lactones reach cancer clinical trials? Drug Discovery Today 2010 15 668 678 10.1016/j.drudis.2010.06.002 20541036

[B10] Ghantous A Tayyoun AA Lteif GA Saliba NA Gali-Muhtasib H El-Sabban M Darwiche N Purified Salograviolide A isolated from Centaurea ainetensis causes growth inhibition and apoptosis in neoplastic epidermal cells International Journal of Oncology 2008 32 841 849 18360711

[B11] Ghantous A Nasser N Saab I Darwiche N Saliba NA Structure–activity relationship of seco-tanapartholides isolated from Achillea falcata for inhibition of HaCaT cell growth European Journal of Medicinal Chemistry 2009 44 3794 3797 10.1016/j.ejmech.2009.04.029 19464086

[B12] El-Najjar N Dakdouki S Darwiche N El-Sabban M Saliba NA Gali-Muhtasib H Anti-colon cancer effects of Salograviolide A isolated from Centaurea ainetensis Oncology Reports 2008 19 897 904 18357373

[B13] Talhouk RS El-Jouni1 W Baalbaki R Gali-Muhtasib H Kogan J Talhouk S Anti-inflammatory bio-activities in water extract of Centaurea ainetensis Journal of Medicinal Plants Research 2008 2 024 033

[B14] Saliba NA Dakdouki S Homeidan F Kogan J Bouhadir K Talhouk S Talhouk R Bio-guided identification of an anti-inflammatory guaianolide from Centaurea ainetensis Pharmaceutical Biology 2009 47 701 707 10.1080/13880200902933021

[B15] Nehmé M Wild flowers of Lebanon 1977 National Council for Scientific Research, Beirut, NCSR Beirut (Lebanon)

[B16] Al-Saghir J Al-Ashi R Salloum R Saliba NA Talhouk RS Homaidan FR Anti-inflammatory properties of Salograviolide A purified from Lebanese plant Centaurea ainetensis BMC Complementary and Alternative Medicine 2009 9 36 10.1186/1472-6882-9-36 19775456 PMC2761300

[B17] Gali-Muhtasib H Fakhoury I Gali-Muhtasib H Salograviolide A: A Plant-Derived Sesquiterpene Lactone with Promising Anti-Inflammatory and Anticancer Effects Advances in Cancer Therapy 2011 InTech, 388 369 388

[B18] Colburn NH Former BF Nelson KA Yuspa SH Tumour promoter induces anchorage independence irreversibly Nature 1979 281 589 591 10.1038/281589a0 492322

[B19] Dhar A Young MR Colburn NH The role of AP-1, NF-kappaB and ROS/NOS in skin carcinogenesis: the JB6 model is predictive Molecular and Cellular Biochemistry 2002 234-235 185 193 12162432

[B20] Huang J Plass C Gerhäuser C Cancer chemoprevention by targeting the epigenome Current Drug Targets 2010 12 1925 1956 21158707 10.2174/138945011798184155

[B21] Huang YW Kuo CT Stoner K Huang TH Wang LS An overview of epigenetics and chemoprevention FEBS Lett 2011 585 2129 2136 10.1016/j.febslet.2010.11.002 21056563 PMC3071863

[B22] Colburn NH Tumor promoter produces anchorage independence in mouse epidermal cells by an induction mechanism Carcinogenesis 1980 1 951 954 10.1093/carcin/1.11.951 11219849

[B23] Dong Z Birrer MJ Watts RG Matrisian LM Colburn NH Blocking of tumor promoter-induced AP-1 activity inhibits induced transformation in JB6 mouse epidermal cells Proceedings of the National Academy of Sciences 1994 91 609 613 10.1073/pnas.91.2.609 PMC42998 8290571

[B24] Weber TJ Siegel RW Markillie LM Chrisler WB Lei XC Colburn NH A paracrine signal mediates the cell transformation response to low dose gamma radiation in JB6 cells Molecular Carcinogenesis 2005 43 31 37 10.1002/mc.20092 15800926

[B25] Li JJ Westergaard C Ghosh P Colburn NH Inhibitors of both nuclear factor-κB and activator protein-1 activation block the neoplastic transformation response Cancer Research 1997 57 3569 3576 9270030

[B26] Young MR Li JJ Rincón M Flavell RA Sathyanarayana BK Hunziker R Colburn NH Transgenic mice demonstrate AP-1 (activator protein-1) transactivation is required for tumor promotion Proceedings of the National Academy of Sciences 1999 96 9827 9832 10.1073/pnas.96.17.9827 PMC22295 10449779

[B27] Hsu TC Nair R Tulsian P Camalier CE Hegamyer GA Young MR Colburn NH Transformation nonresponsive cells owe their resistance to lack of p65/nuclear factor-κB activation Cancer Research 2001 61 4160 4168 11358840

[B28] Watts RG Huang C Young MR Li JJ Dong Z Pennie WD Colburn NH Expression of dominant negative Erk2 inhibits AP-1 transactivation and neoplastic transformation Oncogene 1998 17 3493 3498 10030673 10.1038/sj.onc.1202259

[B29] Hsu TC Young MR Cmarik J Colburn NH Activator protein 1 (AP-1)- and nuclear factor kappaB (NF-kappaB)-dependent transcriptional events in carcinogenesis Free Radical Biology and Medicine 2000 28 1338 1348 10.1016/S0891-5849(00)00220-3 10924853

[B30] Young MR Yang HS Colburn NH Promising molecular targets for cancer prevention: AP-1, NF-[kappa] B and Pdcd4 Trends in Molecular Medicine 2003 9 36 41 10.1016/S1471-4914(02)00009-6 12524209

[B31] Yuspa SH Koehler B Kulesz-Martin M Hennings H Clonal growth of mouse epidermal cells in medium with reduced calcium concentration Journal of Investigative Dermatology 1981 76 144 146 10.1111/1523-1747.ep12525490 7462678

[B32] Greenhalgh DA Welty DJ Player A Yuspa SH Two oncogenes, v-fos and v-ras, cooperate to convert normal keratinocytes to squamous cell carcinoma Proceedings of the National Academy of Sciences 1990 87 643 647 10.1073/pnas.87.2.643 PMC53321 2153961

[B33] Colburn NH Wendel EJ Abruzzo G Dissociation of mitogenesis and late-stage promotion of tumor cell phenotype by phorbol esters: mitogen-resistant variants are sensitive to promotion Proceedings of the National Academy of Sciences 1981 78 6912 6916 10.1073/pnas.78.11.6912 PMC349162 6947266

[B34] Yuspa SH Kilkenny AE Steinert PM Roop DR Expression of murine epidermal differentiation markers is tightly regulated by restricted extracellular calcium concentrations in vitro The Journal of Cell Biology 1989 109 1207 1217 10.1083/jcb.109.3.1207 2475508 PMC2115750

[B35] Suzukawa K Weber TJ Colburn NH AP-1, NF-kappa-B, and ERK activation thresholds for promotion of neoplastic transformation in the mouse epidermal JB6 model Environmental Health Perspectives 2002 110 865 870 12204819 10.1289/ehp.02110865 PMC1240984

[B36] Dong Z Cmarik JL Wendel EJ Colburn NH Differential transformation efficiency but not AP-1 induction under anchorage-dependent and-independent conditions Carcinogenesis 1994 15 1001 1004 10.1093/carcin/15.5.1001 8200060

[B37] Lee KW Kang NJ Heo YS Rogozin EA Pugliese A Hwang MK Bowden GT Bode AM Lee HJ Dong Z Raf and MEK protein kinases are direct molecular targets for the chemopreventive effect of quercetin, a major flavonol in red wine Cancer Research 2008 68 946 955 10.1158/0008-5472.CAN-07-3140 18245498 PMC2276321

[B38] Eferl R Wagner EF AP-1: a double-edged sword in tumorigenesis Nature Reviews Cancer 2003 3 859 868 10.1038/nrc1209 14668816

[B39] Pahl HL Activators and target genes of Rel/NF-kappaB transcription factors Oncogene 1999 18 6853 6866 10.1038/sj.onc.1203239 10602461

[B40] Plati J Bucur O Khosravi-Far R Apoptotic cell signaling in cancer progression and therapy Integative Biology 2011 3 279 296 10.1039/c0ib00144a PMC3130501 21340093

[B41] Hennings H Shores R Wenk ML Spangler EF Tarone R Yuspa SH Malignant conversion of mouse skin tumours is increased by tumour initiators and unaffected by tumour promoters Nature 1983 304 67 69 10.1038/304067a0 6866091

[B42] Ding M Feng R Wang SY Bowman L Lu Y Qian Y Castranova V Jiang BH Shi X Cyanidin-3-glucoside, a natural product derived from blackberry, exhibits chemopreventive and chemotherapeutic activity Journal of Biological Chemistry 2006 281 17359 17368 10.1074/jbc.M600861200 16618699

[B43] Hehner SP Heinrich M Bork PM Vogt M Ratter F Lehmann V Schulze-Osthoff K Dröge W Schmitz ML Sesquiterpene lactones specifically inhibit activation of NF-kappa B by preventing the degradation of I kappa B-alpha and I kappa B-beta Journal of Biological Chemistry 1998 273 1288 1297 10.1074/jbc.273.3.1288 9430659

[B44] Garcia-Pineres AJ Castro V Mora G Schmidt TJ Strunck E Pahl HL Merfort I Cysteine 38 in p65/NF-kappaB plays a crucial role in DNA binding inhibition by sesquiterpene lactones Journal of Biological Chemistry 2001 276 39713 39720 10.1074/jbc.M101985200 11500489

[B45] Van Antwerp DJ Martin SJ Kafri T Green DR Verma IM Suppression of TNF-alpha-induced apoptosis by NF-kappaB Science 1996 274 787 789 10.1126/science.274.5288.787 8864120

[B46] Finco TS Westwick JK Norris JL Beg AA Der CJ Baldwin AS Oncogenic Ha-Ras-induced signaling activates NF-κB transcriptional activity, which is required for cellular transformation Journal of Biological Chemistry 1997 272 24113 24116 10.1074/jbc.272.39.24113 9305854

[B47] Latimer M Ernst MK Dunn LL Drutskaya M Rice NR The N-Terminal Domain of I kappa B alpha Masks the Nuclear Localization Signal (s) of p50 and c-Rel Homodimers Molecular and Cellular Biology 1998 18 2640 2649 9566883 10.1128/mcb.18.5.2640 PMC110643

[B48] Shaulian E Karin M AP-1 as a regulator of cell life and death Nature Cell Biology 2002 4 E131 E136 10.1038/ncb0502-e131 11988758

[B49] Stein B Baldwin AS Ballard DW Greene WC Angel P Herrlich P Cross-coupling of the NF-kappa B p65 and Fos/Jun transcription factors produces potentiated biological function The EMBO journal 1993 12 3879 3891 8404856 10.1002/j.1460-2075.1993.tb06066.x PMC413671

[B50] Angel P Szabowski A Schorpp-Kistner M Function and regulation of AP-1 subunits in skin physiology and pathology Oncogene 2001 20 2413 2423 10.1038/sj.onc.1204380 11402337

[B51] Karin M Lin A NF-kappaB at the crossroads of life and death Nature Immunology 2002 3 221 227 11875461 10.1038/ni0302-221

[B52] Coussens LM Tinkle CL Hanahan D Werb Z MMP-9 supplied by bone marrow–derived cells contributes to skin carcinogenesis Cell 2000 103 481 490 10.1016/S0092-8674(00)00139-2 11081634 PMC2843102

[B53] Schneider-Stock R Ghantous A Bajbouj K Saikali M Darwiche N Epigenetic mechanisms of plant-derived anticancer drugs Frontiers in Bioscience 2012 17 129 173 10.2741/3919 22201736

